# Physical Activity as a Predictor of the Level of Stress and Quality of Sleep during COVID-19 Lockdown

**DOI:** 10.3390/ijerph18115811

**Published:** 2021-05-28

**Authors:** Anna Lipert, Remigiusz Kozłowski, Dariusz Timler, Michał Marczak, Kamila Musiał, Paweł Rasmus, Karolina Kamecka, Anna Jegier

**Affiliations:** 1 Department of Sports Medicine, Medical University of Lodz, 92-213 Lodz, Poland; kamila.musial@umed.lodz.pl (K.M.); anna.jegier@umed.lodz.pl (A.J.); 2 Center of Security Technologies in Logistics, Faculty of Management, University of Lodz, 90-237 Lodz, Poland; remigiusz.kozlowski@wz.uni.lodz.pl; 3 Department of Emergency Medicine and Disaster Medicine, Medical University of Lodz, 92-213 Lodz, Poland; dariusz.timler@umed.lodz.pl; 4 Department of Management and Logistics in Healthcare, Medical University of Lodz, 90-131 Lodz, Poland; michal.marczak@umed.lodz.pl (M.M.); karolina.kamecka@stud.umed.lodz.pl (K.K.); 5 Department of Medical Psychology, Medical University of Lodz, 90-131 Lodz, Poland; pawel.rasmus@umed.lodz.pl

**Keywords:** pandemic, physical activity, quality of sleep, stress in COVID

## Abstract

Background: The coronavirus pandemic and the government restrictions significantly disturbed the daily functioning of people, thereby influencing healthy behaviors, such as physical activity—the core indicator of well-being. This study evaluates the associations between physical activity (PA), the level of stress and quality of sleep during the COVID-19 pandemic lockdown. Methods: An online survey was distributed during the governmental lockdown in April 2020 and included measures for assessing physical activity, stress and sleep. The surveyed participants included all adults aged 18 years and over. The final data were collected from the 1959 respondents using: International Physical Activity Questionnaire-Short Form (IPAQ-SF), Perceived Stress Scale (PSS) and Pittsburgh Sleep Quality Index (PSQI). Findings: Almost half of the respondents indicated a low level of PA, performing only 60 min of PA daily. Most of the participants reported a moderate or high level of stress (57% and 29%, respectively) and 64% of them reported poor quality of sleep. People with low levels of stress performed on average 85.1 min/day of walking (WPA), 40.9 min/day of moderate PA (MPA) or 52.6 min/day of vigorous PA (VPA). People with good quality of sleep performed 82.9 min/day of WPA, 43.6 min/day MPA and 40.5 min/day VPA. Interpretation: The results from the study indicate that the volume of daily PA may be a predictor of the level of stress and sleep quality in adults during the COVID-19 pandemic lockdown. To retain a low level of stress and good quality of sleep, a lifestyle that allows to achieve a moderate level of physical activity should be maintained. The optimal daily dose of PA is at least 70 min per day, involving different intensities.

## 1. Introduction

Since December 2019, the world community has been experiencing a pandemic from a novel coronavirus SARS-CoV-2, known as COVID-19 [1,2]. The scale of the pandemic has resulted in the worldwide concern over the extent to which human health and well-being have been affected as a result of the changes in people’s everyday life [3], limiting the opportunities to perform physical activity (PA).

Physical activity is at the core of health and well-being and the benefits of PA, such as lower levels of stress, anxiety and depressed mood, are well-known. With the enactment of social isolation and physical distancing restrictions and other actions aimed to reduce the COVID-19 pandemic, the usual places for physical activities, e.g., gyms and outdoor recreation facilities, were no longer accessible. Although there are people who have their own strong inner need for physical activity, most of the population probably reduced their PA due to the absence of social support or concerns for contracting the virus in an outdoor environment [3].

During the COVID-19 pandemic, there has been widespread emotional distress caused by the outbreak itself or the measures taken to bring it under control [4,5]. Moreover, many people changed the form of fulfilling their work obligations or even lost their source of income, which may additionally augment psychological distress for both men and women [6]. Data from China suggests that about 25% of the general population have experienced moderate to severe levels of stress- or anxiety-related symptoms in response to COVID-19 [7,8]. Facing stressful situations may cause symptoms such as sleep suppression and increased wakefulness, thus increasing the occurrence of insomnia, daytime sleepiness, nightmares and daytime dysfunction [9]. Additionally, sleep disturbances are prevalent, especially in individuals who are forced into medical isolation [10].

Sleep is an indispensable physiological process in maintaining physical health [3] and an integral part of proper human functioning [11]. Having a good quality of sleep is important in strengthening immunity [12], hence any sleep disturbances being a consequences of stress induced by the COVID-19 pandemic may increase susceptibility to infection or compromise recovery in the event of an infection [13].

Early evidence gathered during the COVID-19 outbreak suggests positive associations between increased physical activity, physical health [3] and lower psychological distress [14,15]. However, more detailed exploration of the COVID-19 pandemic lockdown and thus restricted physical activity and its impact on health may help direct future public health policy to maintain well-being of the community. Therefore, the present study aims to evaluate associations between the volume of physical activity and the level of stress and sleep quality due to the implementation of social isolation rules during the COVID-19 pandemic lockdown.

## 2. Material and Methods

### 2.1. Study Design and Data Collection

Data was collected form 1959 respondents. An anonymous online survey was hosted on the survey platform Google and distributed using social media sources (Facebook, Twitter), with the help of popular national Influencers via their web pages and via institutional sources, including email. The surveyed participants included all adults aged 18 years and over. Participation in the study was voluntary and did not involve any form of gratification. Completing the questionnaire was anonymous and equivalent to agreeing to participate in the study, so the relevant University Human Ethics Committee decided to exempt it from the obligation of their approval. Data collection occurred during the governmental lockdown between 1 and 14 April 2020. The study meets the ethical standards of the journal.

At the time of the survey distribution, the situation in the country required significant personal distancing, lockdown and travel restrictions. No person was allowed to leave the house except for a journey to work or necessary shopping. People under 18 years old were allowed to leave the house only under the supervision of an adult. Social distancing measures included keeping a minimum 2 m distance between people, a ban on any public gatherings and a limited number of people in the shops and on public transport. There were also designated hours, between 10 and 12 am, for seniors in the shops when nobody under 65 years of age was allowed to enter. Lockdown restrictions also included the closure of restaurants and bars, hotels, parks and beaches, hairdressers and beauty studios. Schools and universities were closed, with face-to-face teaching transitioned to online learning. Travel within towns and cities was only permitted for essential work or workers, or to access essential services such as medical or healthcare.

Socio-demographic data was collected using a self-made questionnaire in which the questions were selected to get the relevant information influencing primarily the physical activity level, the quality of sleep and stress level.

The physical activity (PA) level was self-reported using the International Physical Activity Questionnaire-Short Form (IPAQ-SF), which estimates the last 7 days’ activity [16,17]. IPAQ-SF comprises items assessing the frequency and duration of physical activity in three ranges of intensity: vigorous physical activity (VPA = 8.0 metabolic equivalent (METs)), moderate physical activity (MPA = 4.0 METs) and low physical activity, determined as walking (WPA = 3.3 METs) undertaken across a set of domains, including leisure time, household activities and gardening (yard), and work-related and transport-related activities during a typical week of their life. The final results of IPAQ were proceeded in accordance with the IPAQ scoring protocol guideline [17,18] and presented as the total count of minutes of physical activity per day.

The quality and patterns of sleep for adults was measured by the Pittsburgh Sleep Quality Index (PSQI). It differentiates “poor” from “good” sleep quality by measuring the following seven areas (components): subjective sleep quality, sleep latency, sleep duration, habitual sleep efficiency, sleep disturbances, use of sleeping medications and daytime dysfunction over the last month. A total score of “5” or more is indicative of poor sleep quality. The evidence synthesis for the PSQI showed its strong reliability and validity in a variety of samples, suggesting that the tool fulfils its intended utility [19].

The level of stress was measured by the Perceived Stress Scale (PSS), which is a validated [20,21] classic stress assessment instrument originally developed in 1983. The questions in this scale refer to feelings and thoughts during the last month. Individual scores on the PSS can range from 0 to 40, with higher scores indicating higher levels of perceived stress: the range between 0 and 13 points means a low level of stress (LLS), the score between 14 and 26 points indicates a moderate level of stress (MLS) and the range of 27–40 points means a high level of stress (HLS).

### 2.2. Statistical Analysis

Statistical analyses were performed using the STATISTICA (StatSoft, Inc. 2011, Statsoft Polska Sp. z o.o., Lodz, Poland, version 10, www.statsoft.com, accessed on 23 March 2015). When the Shapiro–Wilk test revealed that data were normally distributed, parametric tests were performed and the statistical analysis was performed by Student’s *t*-test. When data was not normally distributed, non-parametric tests were used. The analysis of variance (ANOVA) was also performed to observe the relationship between time of PA, the level of stress and the quality of sleep. To compare the percentage of categorical variables (level of PA, level of stress, quality of sleep), the chi-square test was performed. The relationship between physical activity and stress (PA min/day and PSS score) was conducted using the Pearson correlation. All confidence intervals (CIs) are presented as 95% CI. Significant differences were accepted for all analyses at the level of *p* < 0.05.

## 3. Results

### 3.1. Characteristics of the Participants

**Figure 1:**
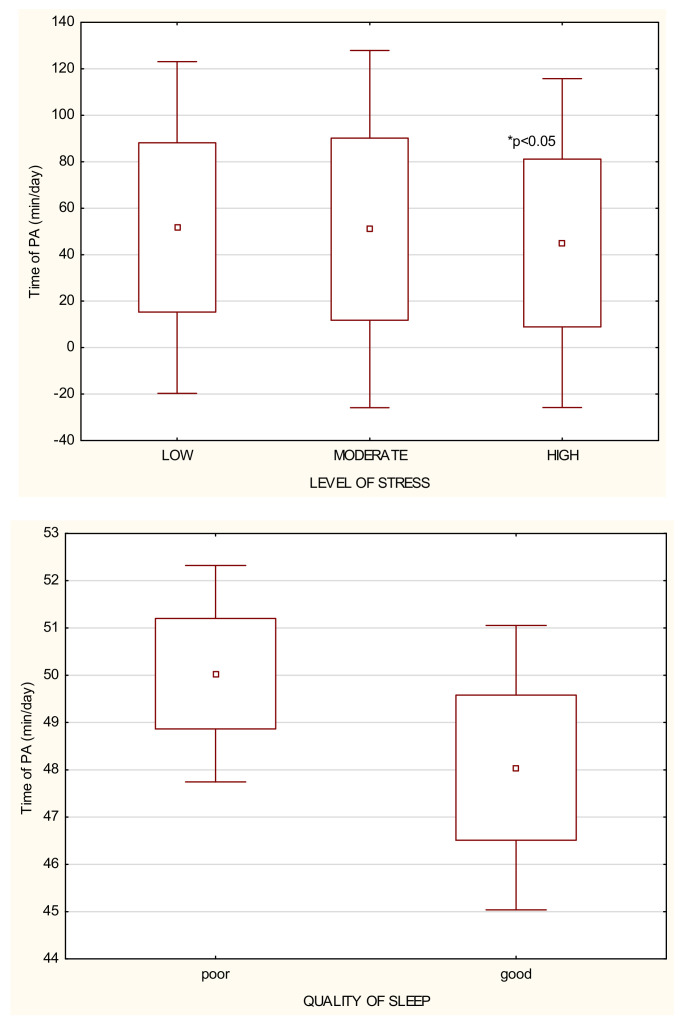
The relationship between the time of PA per day performed by the study participants (*n* = 1956), the level of stress and the quality of sleep.

The general characteristics of the study participants can be found in Table 1, Table 2 and Table 3 and Figure 1. A large group of the participants was represented by people living in a city of over 100,000 citizens and declaring that they performed office work. However, half of them did not work during the pandemic for such reasons as vacation, dismissal or job loss. Most of the study participants had to comply with the restrictions resulting from government regulations. The study participants reported their habitual physical activity of 49.32 ± 38.01 min/day on average. Almost half of the study participants had a low level of PA, being physically active for only 24.30 ± 16.74 min/day in comparison to people with moderate, 69.82 ± 23.82 min/day, and high levels of PA, 116.12 ± 27.19 min/day, with *p* < 0.001 (Table 3 and Figure 1). Generally, most of the study participants were characterized by moderate or high levels of stress and usually poor quality of sleep (Table 2).

### 3.2. Level of Stress in Relation to Physical Activity during Lockdown

**Figure 2:**
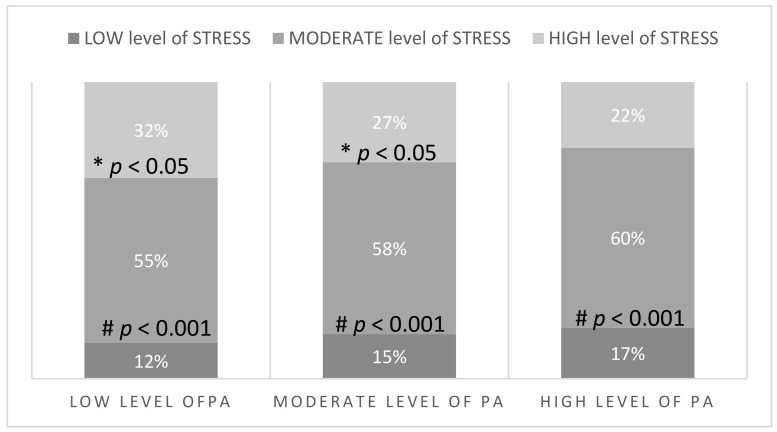
Number and the percentage of participants (*n* = 1956) with a specific level of stress depending on the level of PA. * *p*-value LOW vs. HIGH across different level of PA groups, # *p*-value MODERATE vs. LOW and HIGH across different level of PA groups.

**Figure 3:**
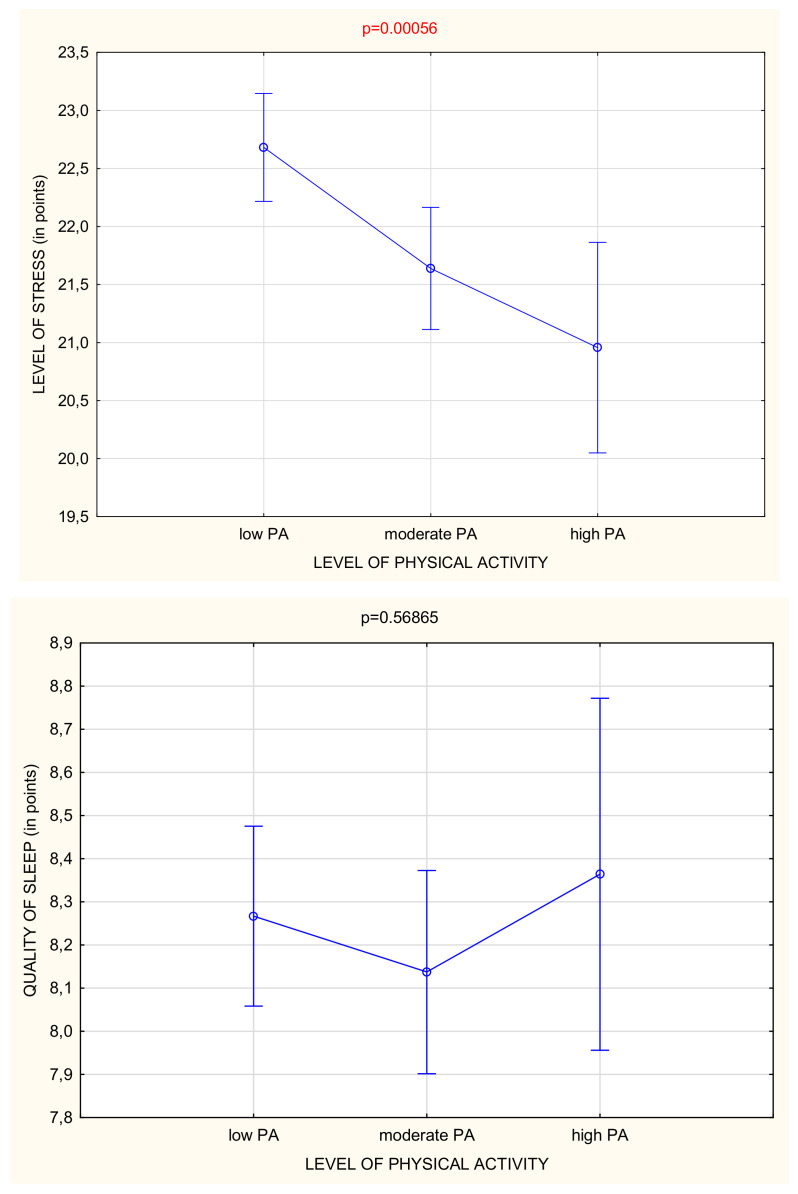
Level of stress and quality of sleep depending on the level of PA.

In the whole study group, most of the participants had a moderate or high level of stress (Table 2). However, there were fewer people with a high level of stress among those who were classified as moderately or highly active (Figure 2). People with the high level of stress performed physical activity for only 44.99 ± 36.10 min/day, while people with moderate and low levels of stress performed 50.99 ± 39.21 and 51.71 ± 36.43 min/day, respectively (Figure 1 and Table 4). Generally, it was observed that if the level of physical activity was higher, the stress level was significantly reduced (*p* < 0.001) (Figure 3). 

### 3.3. Quality of Sleep in Relation to Physical Activity during Lockdown

**Figure 4:**
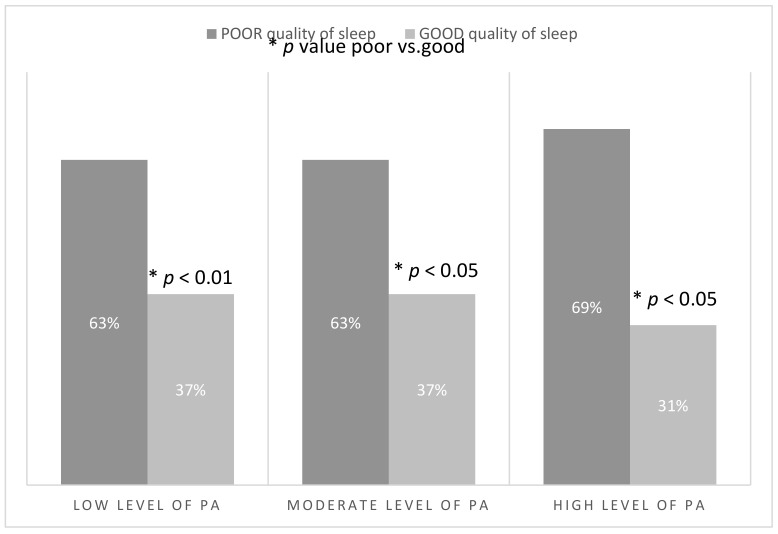
Number and the percentage of participants (*n* = 1956) with a specific quality of sleep depending on the level of PA.

Over a half of the study participants reported poor quality of sleep (Table 2). People who declared a poor quality of sleep performed PA for 50.03 ± 38.24 min/day on average, in comparison to those with good quality of sleep who undertook on average 48.04 ± 37.60 min/day (Table 5). No difference was observed in the number of people with low and moderate levels of PA declaring a good quality of sleep, but there were also many highly active people with poor quality of sleep (Figure 4). It was observed that if the level of physical activity was moderate, the quality of sleep was better. However, too much physical activity can influence the quality of sleep negatively (Figure 3).

### 3.4. Optimal Time and Intensity of PA during Lockdown

Table 4 and Figure 3 show that the more time was spent on physical activity, the less stress the study participants experienced. However, it was also observed that too much PA during the day affected the quality of sleep (Figure 3). The linear correlation −0.523 also confirmed that there is a moderate negative relationship between PSS scores and the time of PA. It seems that the most favorable is a moderate level of physical activity, or performing about 70 min/day of habitual PA of different intensities (Table 3) to reduce stress and have a good quality of sleep (Figure 3). Apart from the amount of time devoted to physical activity, the intensity of the PA was also an important factor. There was no similar relationship observed for sleep quality (Table 4).

## 4. Discussion

Prolonged, harmful stress related to quarantine and limited social and physical contact with others could be a cause of serious mental disorders [22,23]. During the COVID-19 pandemic lockdown, the access to activities which decrease stress level such as work activities, interpersonal contact, physical activity or other hobbies was limited due to government restrictions implemented by governments in many countries. To our knowledge, this study was one of the first to have examined associations between stress, quality of sleep and limited physical activity after the implementation of social isolation rules during the COVID-19 pandemic lockdown.

The results of the study showed a high level of stress in most of the study participants. There are several reasons for stress during a pandemic, including the fear of the unknown, anxiety, prolonged uncertain situation, quarantine and limited contact with relatives and friends, lack of vaccines and medications for the virus, fear and high risk of contracting the virus, either by oneself or by family members, and also financial loss [23,24]. Studies in China [9,25] during lockdown due to the COVID-19 pandemic evidenced that the intensity of the anxiety symptoms was associated with the worry about economic situation and stability of the family income. The enhanced perceived stress could also be explained by the fact that people who did not work during the pandemic were more exposed to information in the media, most of which was related to the pandemic, which contributed to accumulation of disturbing thoughts about the COVID-19 outbreak and its consequences [26] or fear of falling ill. It confirms that PA helps to decrease emotional and physical tension [7,27,28], which leads to a decrease in stress level even during as stressful situation as the COVID-19 pandemic.

In the present study, most of the participants proved to have poor quality of sleep (QS). Sleep problems could be explained by the working time which drastically changed, especially for people working remotely because they work became quite irregular and dependent on the domestic environment. The influence of working time on sleep quality can be observed among healthcare workers who face the challenge of severe epidemic and insufficient time for rest, which results in chronic stress and psychological distress [26] and, consequently, to sleep disturbances. Poor quality of sleep can be related to stress as well as to circadian rhythm disruption, increasing the risk of severe metabolic disorders [29]. Additionally, the accumulation of intensive thinking about the COVID-19 pandemic could, in turn, lead to difficulties in falling asleep and a decrease in QS. However, again, the study participants who were engaged in physical activity reported their quality of sleep to be better than the inactive ones. A previous study demonstrated a correlation between sleep and physical activity [30] and revealed that regular PA improves QS [30].

One of the available studies showed that during lockdown, the study participants were inactive and spent most of their time watching TV or using other media tools [3,24,31]. An Italian study showed that energy expenditure on PA was significantly lower than before the pandemic in all age groups, especially among men [32]. A decrease of PA level was visible, especially among people with a high pre-pandemic activity level [33]. Other studies showed that the COVID-19 pandemic caused a decrease in PA level of each intensity (vigorous, moderate, walking and overall) and resulted in increased daily sitting time [34]. The decline of physical activity could be accounted for by social distancing, travel restriction, closure of gyms, swimming pools, amusement parks and green areas, i.e., places where PA was commonly performed prior to the COVID -19 pandemic [3]. A sudden PA level reduction could lead to numerous negative health consequences, especially metabolic diseases, cardiovascular diseases [35] and obesity, but could also cause mental disorders such as anxiety and depression [36] which altogether can increase the risk of COVID-19 infection and poor prognosis [37]. Some studies confirm that only regular, vigorous PA could decrease a high level of anxiety during the COVID-19 pandemic [38]. The present study revealed a low level of PA in almost half of the study participants.

To sum up, the study showed a lower SL and better QS during the COVID-19 pandemic lockdown in the participants who were engaged in PA. It should be recommended to undertake even the simplest forms of physical activity and use mobile technologies such as telephone applications and wearable sensors to encourage exercise [39]. There should be a moderate level of habitual PA obtained lasting about 70 min/day, and composed of PA of different intensities. The most beneficial duration of physical activity seems to be 40 min/day, as a combination of walking as well as moderate or vigorous PA.

There are a number of strengths of the present study, including a large sample size, the timing of data collection related to the lockdown in Poland and the usage of 3 internationally recognized tools [17,19,20]. However, some limitations should be acknowledged. Firstly, all the data are self-reported, which means that the responses are subject to recall bias, but it was the only manageable form of data collection during the pandemic-related restrictions [3,4]. There is socio-demographic information about the participants with no anthropometric and clinical data, but anthropometric data would have to be measured in direct contact, which was also impossible during the lockdown. Regarding the clinical data, there may have been concerns about giving too much medical data in an online questionnaire rather than in personal contact to the interviewer. Secondly, there was a higher response rate among women, however, most surveys conducted during the COVID-19 pandemic suffered from an overrepresentation of the female gender [4,40] as women appear to be more willing to complete this type of survey. Lastly, it was a cross-sectional observational study and more longitudinal data are needed to observe changes over time to assess the impact of social restrictions and social distancing on human health.

## 5. Conclusions

Our data suggest that the volume of physical activity can be a predictor of the level of stress and quality of sleep of adults during the COVID-19 pandemic lockdown. To retain a low level of stress and good quality of sleep, a lifestyle that allows to achieve a moderate level of physical activity should be maintained. The volume of recommended PA should be at least 70 min per day, involving different intensities. During the future pandemic restrictions, special attention should be paid to physical activity recommendations to improve the health status and well-being of society.

## Figures and Tables

**Table 1:** The main socio-demographic variables characterizing the study group (*n* = 1959).

Socio-Demographic Variables	All the Study Group *n* (%)
**Sex**	
Female	1681 (85.8)
Male	278 (14.2)
**Place of living**	
City > 100,000 citizens	999 (51.0)
City 20–100,000 citizens	379 (19.3)
Town < 20,000 citizens	180 (9.2)
Village	401 (20.5)
**Type of work**	
Office work	1149 (58.6)
Physical work	445 (22.7)
Not applicable	366 (18.7)
**Occupational status**	
Unemployed	421 (21.5)
Employed	1159 (59.1)
Retirement	20 (1.0)
Student	359 (18.4)
**F** **orm of work during pandemic**	
Not working/unemployed	976 (49.8)
Remote work	639 (32.6)
In the workplace	344 (17.6)
**Form of lockdown**	
Governmental restrictions	1538 (78.5)
Quarantine	23 (1.2)
**N** **o restrictions because of the type of occupation (medical staff, etc.)**	398 (20.3)

**Table 2:** The characteristics of the study participants regarding the volume of PA, level of stress and quality of sleep (*n* = 1959).

	All the Study Group *n* (%)
**Level of PA during pandemic lockdown**	
Low PA	957 (49)
Moderate PA	750 (38)
High PA	252 (13)
**Level of stress during pandemic lockdown**	
Low (LLS)	277 (14)
Moderate (MLS)	1118 (57)
High (HLS)	564 (29)
**Quality of sleep during pandemic lockdown**	
Poor	1256 (64)
Good	703 (36)

LLS—low level of stress; MLS—moderate level of stress; HLS—high level of stress.

**Table 3:** Time of physical activity, level of stress and quality of sleep in the study population (*n* = 1956) according to the level of PA.

	All the Study Group	Low Level of PA	Moderate Level of PA	High Level of PA
	Mean (±SD)			
Total PA (min/day)	49.32 ± 38.01	24.30 ± 16.74 *	69.82 ± 23.82 ^#^	116.12 ± 27.19
Walking PA (min/day)	30.82 ± 33.01	16.00 ± 16.25	42.06 ± 35.63	65.77 ± 41.73
Moderate PA(min/day)	7.98 ± 9.66	4.98 ± 7.62	11.23 ± 10.50	12.29 ± 10.81
Vigorous PA (min/day)	9.57 ± 12.14	2.96 ± 5.22	15.93 ± 12.85	18.71 ± 14.76
PSSQ (in points)	22.06 ± 7.36	22.68 ± 7.37	21.63 ± 7.25	20.96 ± 7.47
GPQS (in points)	8.23 ± 3.29	8.26 ± 2.71	8.13 ± 2.74	8.36 ± 5.82

* *p* < 0.001 Low vs. High; ^#^ *p* < 0.05 Moderate vs. High.

**Table 4:** Time of physical activity according to the level of stress in the study population (*n* = 1956).

PA and Stress	Low Level of Stress	Moderate Level of Stress	High Level of Stress
	Mean (±SD)		
Total PA (min/day)	51.71 ± 36.43	50.99 ± 39.21	44.99 ± 36.10 *
Walking PA (min/day)	29.83 ± 30.47	31.99 ± 34.09	29.01 ± 31.99
Moderate PA(min/day)	8.93 ± 10.30	8.34 ± 9.69	6.82 ± 9.20 *
Vigorous PA (min/day)	10.97 ± 12.55	9.72 ± 11.99	8.60 ± 12.18 *

* *p* < 0.05 High vs. Low and Moderate.

**Table 5:** Time of physical activity according to the quality of sleep in the study population (*n* = 1956).

PA and Sleep Quality	Poor Sleep Quality	Good Sleep Quality
	Mean (±SD)	
Total PA (min/day)	50.03 ± 38.24	48.04 ± 37.60
Walking PA (min/day)	30.92 ± 33.18	30.63 ± 32.73
Moderate PA (min/day)	8.18 ± 9.68	7.61 ± 9.61
Vigorous PA (min/day)	9.94 ± 12.27	8.91 ± 11.89

## Data Availability

The data presented in this study are available on request from the corresponding author.

## Correction Statement

The Editorial Office and Editorial Board would like to inform readers of a post-publication review process that was conducted related to this publication. Following concerns about a potential conflict of interest between the authors and the original Academic Editor, the Editorial Office has conducted a post-publication peer-review of this article. This process included the recruitment of a new Academic Editor to ensure full compliance with MDPI’s Editorial Process (https://www.mdpi.com/editorial_process). As a result of this process, the original Academic Editor was removed, and a new Academic Editor is listed. The new Academic Editor is satisfied that the editorial process for this article has been completed in accordance with MDPI’s Editorial Process policy.
